# Genetic Dissection of Shelling Percentage in Maize via QTL Mapping Using a Maize-Teosinte Population

**DOI:** 10.3390/genes17040384

**Published:** 2026-03-28

**Authors:** Yan Bai, Yifei Wang, Xiangyin Hou, Jinsheng Lai, Weibin Song

**Affiliations:** 1 State Key Laboratory of Maize Bio-Breeding, Key Laboratory of Maize Biology and Genetic Breeding, Key Laboratory of Genome Editing Research and Application, Ministry of Agriculture and Rural Affairs, National Maize Improvement Center, China Agricultural University, Beijing 100193, China; by271039957@163.com (Y.B.); 18375730586@163.com (Y.W.); xiangyinhou@cau.edu.cn (X.H.); jlai@cau.edu.cn (J.L.); 2 National Agricultural Technology Extension & Service Center, Beijing 100125, China; 3 Center for Crop Functional Genomics and Molecular Breeding, China Agricultural University, Beijing 100193, China; 4 International Maize Research Center, Sanya Institute of China Agricultural University, Sanya 572024, China

**Keywords:** maize, shelling percentage, QTL mapping

## Abstract

**Background:** Shelling percentage is an important trait affecting grain yield efficiency in maize, but its genetic basis remains insufficiently understood. **Methods:** In this study, a maize-teosinte BC_2_S_2_ population derived from Zheng58 and (*Zea mays* ssp. *parviglumis*, CIMMYTMA 8782) was used for phenotypic evaluation and QTL mapping of shelling percentage across two replicates and BLUP-based analysis. Candidate genes were further prioritized based on their positions within QTL support intervals, expression patterns, and functional annotation. **Results:** Two reproducible QTLs, *qSP7* and *qSP10*, were identified on chromosomes 7 and 10, respectively. *qSP7* explained 3.39–3.41% of the phenotypic variation, whereas *qSP10* explained 2.96–6.45%. Within these intervals, *Zm00001d021701*, *Zm00001d021708*, and *Zm00001d025739* were prioritized as candidate genes based on expression and annotation evidence. **Conclusions:** These results indicate that shelling percentage in maize is controlled by multiple loci with modest effects and provide a basis for future genetic analysis and marker development for this trait.

## 1. Introduction

Maize (*Zea mays* L.) is one of the world’s most important crops and plays a vital role in ensuring food security. Increasing grain yield remains an enduring goal in maize breeding. Maize yield is determined by three key factors: number of ears per unit area, number of kernels per ear, and hundred-kernel weight (HKW). Shelling percentage is defined as the ratio of total dry weight of kernels to total dry weight of the ears [1,2]. A higher shelling percentage indicates greater efficiency in allocating biomass to kernels along the cob. This trait is central to maize domestication and modern breeding programs, and improving shelling percentage is essential for increasing overall maize production.

Currently, studies directly targeting QTL mapping for shelling percentage are scarce [3,4]. Since researchers pay little attention to cob yield, they focus more on traits that directly influence grain yield, such as kernel length, kernel width, kernel thickness, and kernel weight. Although extensive research has been conducted on QTL mapping for kernel-related traits [5,6,7,8], most studies remain confined to identifying broad QTL intervals. The number of maize QTLs associated with kernel traits that have been successfully cloned remains limited. *ZmKW1* encodes an E3 ubiquitin ligase, which is highly expressed in the endosperm region and negatively regulates kernel size and weight. Through fine-mapping and candidate gene association analysis, an Indel-1346 located 2.8 kb upstream of the *ZmKW1* promoter was identified to enhance gene expression. Carrying the Indel-1346 locus reduces maize kernel weight [9]. *ZmBAM1d* is the functional gene for the *qHKW1* locus controlling maize kernel shape and weight. This gene positively regulates kernel weight, and its overexpression or knockout has no effect on other agronomic traits [10]. The functional gene of kernel weight, QTL-*qKW9*, encodes a PPR protein with a DYW domain, which participates in C-U editing of the chloroplast NADH dehydrogenase-like complex subunit ndhB. Mutations in this gene reduce photosynthetic capacity, decrease photosynthetic products available for kernel filling, and ultimately affect kernel weight [11]. *ZmEXPB15* regulates maize kernel weight, through natural population association analysis, researchers identified a site in the gene promoter region significantly associated with hundred-kernel weight, with its expression level showing a significant positive correlation with hundred-kernel weight. This gene synergistically regulates early maize kernel development with *ZmNAC11* and *ZmNAC29*, significantly increasing kernel weight by accelerating endosperm tissue elimination [12]. On maize chromosome 9, the major QTL for kernel length, *KL9*, was also identified. *ZmKL9* encodes a bZIP transcription factor. A transposable element insertion–deletion in its 5′UTR region constitutes a functional site causing kernel size variation. This gene positively regulates maize kernel size and yield [13]. Many kernel-related genes were cloned from mutants such as defective kernel, empty pericarp, and small kernel, which typically develop small and/or lethal kernels with little practical value [14,15,16,17,18]. Maize kernel size is regulated by multiple molecular pathways, primarily involving G protein signaling, the ubiquitin–proteasome system, the MAPK signaling pathway, and the growth hormone pathway [19,20]. In recent years, several kernel-related genes have been identified through reverse genetics approaches. These genes encode diverse proteins, including transcription factors, enzymes, and miRNAs [21,22,23,24,25], indicating that kernel traits are governed by complex genetic networks. Despite extensive research on numerous kernel-related genes in maize, the number of identified kernel-related QTLs remains limited. Further exploration of kernel-related QTLs is needed to provide genetic resources for the genetic improvement of maize kernel traits.

Maize was domesticated from its wild ancestor teosinte (*Zea mays* ssp. *parviglumis*). Although teosinte lacks a cob structure, its kernels possess a hard, dense lignin glume controlled by the *Tga1* gene [26]. The glume of teosinte seeds constitutes a non-seed component accounting for a significant portion of the mass and should be included as the denominator in shelling percentage. This characteristic results in a relatively low shelling percentage for teosinte.

To dissect the genetic basis of shelling percentage in maize, we developed a maize–teosinte BC_2_S_2_ population with strong phenotypic contrast for this trait and conducted QTL mapping, followed by candidate gene analysis within the detected QTL intervals.

## 2. Materials and Methods

### 2.1. Plant Materials and Field Trials

A maize–teosinte mapping population was developed using the elite maize inbred line Zheng58 as the recurrent parent and teosinte (*Zea mays* ssp. *parviglumis*, CIMMYTMA 8782) as the donor parent. After two rounds of backcrossing to Zheng58 followed by two generations of selfing, a BC_2_S_2_ population was generated, hereafter referred to as the TM population. A total of 590 BC_2_S_2_ lines were used in this study. Population development and genotyping were completed in 2022, whereas phenotypic evaluation for shelling percentage was conducted in 2025.

The TM population and the two parental lines were grown in Gongzhuling, Jilin Province, China (43°30′ N, 124°49′ E) in 2025 with two field replicates. Each line was planted in a single-row plot containing 13 plants, with 60 cm between rows and 25 cm between adjacent plants within a row. Standard local field management practices were applied throughout the growing season.

At physiological maturity, ears from each plot were harvested and naturally dried before measurement. For each plot, at least five well-filled ears were selected for shelling percentage evaluation. Ears with poor grain filling or obvious defects were excluded. Kernels were manually separated from the cob, and the dry weights of kernels and cobs were recorded separately. Shelling percentage was calculated as Shelling percentage = kernel dry weight/ear dry weight, where ear dry weight was defined as the sum of kernel dry weight and cob dry weight.

### 2.2. Phenotypic Analysis

Phenotypic data for shelling percentage from the two field replicates were summarized using descriptive statistics in Microsoft Excel 2019 (Microsoft, Redmond, WA, USA). Trait distribution and correlation analyses were performed in R (R Foundation for Statistical Computing, Vienna, Austria). Pearson correlation coefficients between the two replicates were calculated using the Performance Analytics package (v2.0.4). Broad-sense heritability (*H*^2^) was estimated as *H*^2^ = σg^2^/(σg^2^ + σe^2^/n), where σg^2^ is the genetic variance, σe^2^ is the residual variance, and n is the number of replicates [27,28].

To obtain an integrated phenotypic estimate across replicates, best linear unbiased prediction (BLUP) values were calculated using a mixed linear model implemented in the lme4 package (v1.1–27.1) in R, with genotype and replicate treated as random effects [27,28]. The BLUP values, together with the raw phenotypic data from the two field replicates, were used for subsequent QTL analyses.

### 2.3. Linkage Map Construction and QTL Identification

Genotype data for the BC_2_S_2_ TM population were obtained by genotyping-by-sequencing (GBS). The average sequencing depth was 0.1× for each line. Raw sequencing reads were first processed using fastp (v0.23.2) for quality control and adapter trimming, and the filtered reads were aligned to the B73 reference genome version 4 (B73 RefGen_v4) using BWA (v0.7.17) [29,30]. Variant calling was performed with GATK HaplotypeCaller (v4.2.0.0) [31], and individual GVCF files were combined for joint genotyping to generate a multi-sample VCF file.

To obtain a high-quality SNP dataset, variants were filtered using the following criteria: those with a minor allele frequency (MAF) < 0.05, marker missing rate > 40%, and individual missing rate > 50% were excluded. The filtered markers were then used for linkage map construction.

A genetic linkage map was constructed in R/qtl (v1.50). Missing genotypes were imputed using the fill.geno function, and recombination fractions were converted to genetic distances using the Kosambi mapping function implemented in est.map. QTL mapping for shelling percentage was conducted by composite interval mapping (CIM) using the phenotypic data from the two field replicates and the BLUP values [32].

Genome-wide significance thresholds were determined by 1000 permutation tests for each dataset. QTL support intervals were defined using the 1.5-LOD drop method. QTLs detected in overlapping genomic regions across multiple datasets were considered reproducible QTL signals.

### 2.4. Candidate Gene Analysis

Candidate gene analysis was conducted for the QTL intervals based on the B73 RefGen_v4 genome annotation. All annotated genes within the corresponding support intervals were first retrieved as the initial candidate set.

To reduce the candidate list, expression information for these genes was first examined using qTeller (MaizeGDB). Expression in V18 immature cob and whole seed at 22 days after pollination (22 DAP) was used as the initial screening criterion because shelling percentage is closely related to the relative contribution of cob and kernel tissues to ear biomass. Genes with expression values below 5 FPKM in both tissues were excluded from subsequent analysis. The genes retained after this initial filtering were then further examined using publicly available maize transcriptome data [33]. Relative expression patterns across nine tissues (kernel, cob, ear, tassel, leaf, root, silk, anther, and shoot apical meristem) were visualized by heatmap analysis.

Finally, candidate genes were prioritized according to their physical positions within the QTL support intervals, their expression patterns in relevant tissues, and their annotated or reported functions related to kernel development, cob development, biomass allocation, or regulatory pathways potentially associated with shelling percentage.

## 3. Results

### 3.1. Phenotypic Evaluation

**Figure 1:**
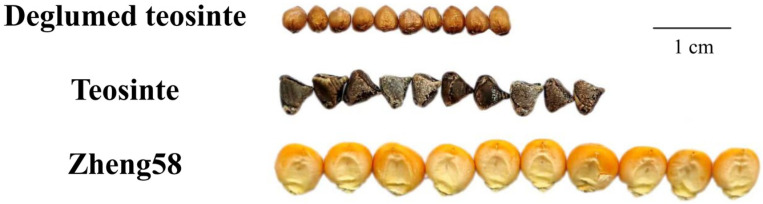
Kernel phenotypes of the two parental lines used for the construction of the TM population. Zheng58 and teosinte showed clear differences in kernel morphology. A scale bar is included to facilitate visual comparison between the two parents.

**Figure 2:**
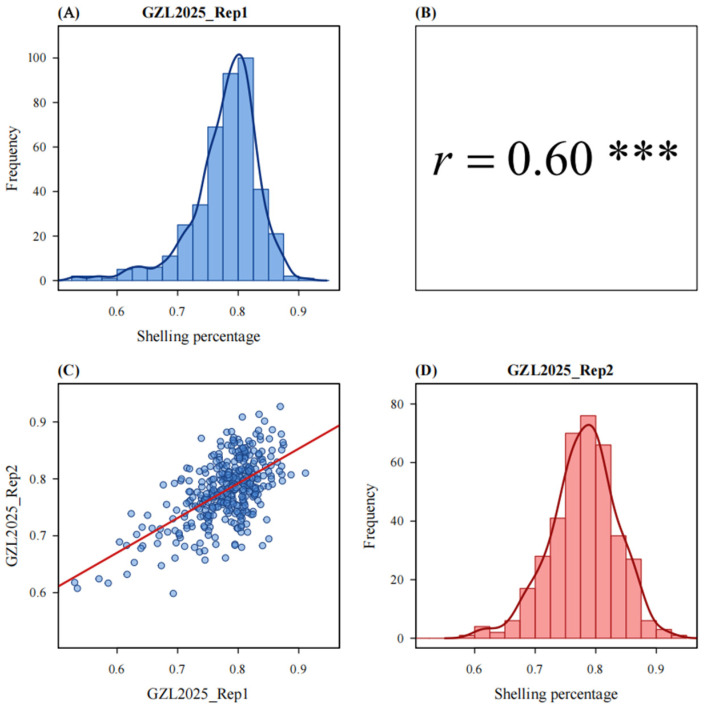
Phenotypic distribution of shelling percentage and correlation between two field replicates in the TM population. (**A**) Frequency distribution of shelling percentage in GZL2025_Rep1. (**B**) Pearson correlation coefficient between GZL2025_Rep1 and GZL2025_Rep2. (**C**) Scatter plot showing the relationship between shelling percentage values measured in GZL2025_Rep1 and GZL2025_Rep2; each point represents one BC_2_S_2_ line, and the red line indicates the fitted linear regression. (**D**) Frequency distribution of shelling percentage in GZL2025_Rep2. The shelling percentage showed an approximately continuous distribution in both replicates, consistent with quantitative inheritance. The correlation between the two replicates was significant (*r* = 0.60; *** *p* < 0.001).

As shown in Figure 1, the kernel morphology of the parental lines used in this study. Teosinte (CIMMYTMA 8782) possesses hard glumes that account for a significant portion of its mass, whereas Zheng58 lack such hard glumes. As shown in Table S1, the shelling percentage trait exhibits a high heritability of 0.75. In GZL2025_Rep1, the range of shelling percentage across populations was 0.53–0.91, while in GZL2025_Rep2, it ranged from 0.60 to 0.93. The mean shelling percentage across both replicates was 0.79. The phenotypic distributions for the two replicates are shown in Figure 2, with a Pearson correlation coefficient of 0.6.

### 3.2. Construction of Linkage Map and Physical Map

**Figure 3:**
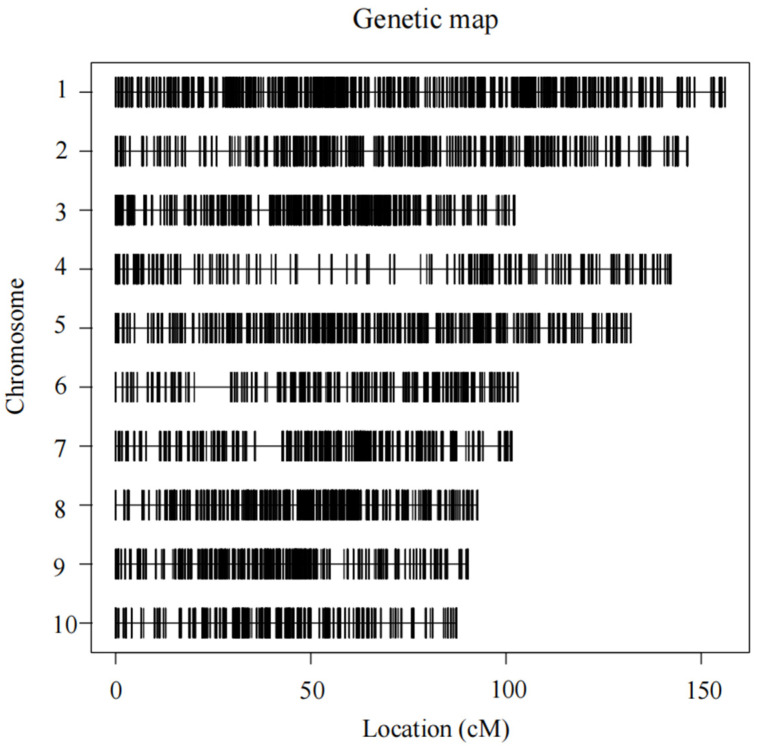
**Genetic linkage map of the TM population.** The map includes 10 maize chromosomes, with each horizontal line representing one chromosome and each vertical tick mark indicating the position of a mapped marker. Marker positions are shown in centimorgans (cM) along the *x*-axis. The distribution of markers across chromosomes illustrates the overall coverage and density of the linkage map used for QTL detection in this study.

The TM population genetic map is shown in Figure 3, with detailed statistical data presented in Table S2. The total chromosomal genetic distance spans 1159.82 cM, comprising 10,329 SNP markers. Among these, chromosome 1 exhibits the longest genetic distance at 156.56 cM, while chromosome 10 has the shortest at 88 cM. Chromosome 1 contains the highest number of SNP markers (1916), while chromosome 4 has the fewest (632). The maximum physical distance between adjacent markers is 15.69 Mb, and the minimum is 3.568 Mb. The average physical distance between adjacent SNP markers across all ten chromosomes ranges from 0.16 Mb to 0.39 Mb.

### 3.3. Identification of QTL for Shelling Percentage

**Figure 4:**
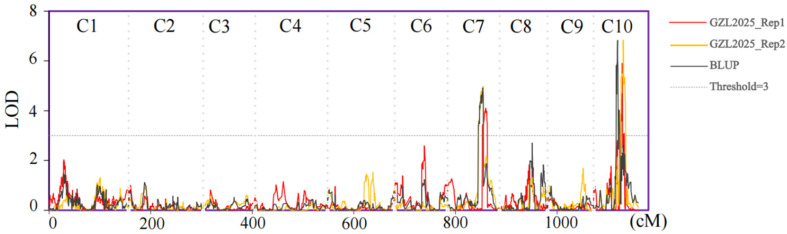
QTL mapping results for shelling percentage in the TM population across 3 replicates.

We conducted QTL mapping for shelling percentage in the TM population, including two replicates and BLUP, treated as three replicates. All three scans identified two shelling percentage QTLs (*qSP7* and *qSP10*) located on chromosomes 7 and 10, respectively, as shown in Figure 4. *qSP7* explained 3.39–3.41% of phenotypic variation across the three replicates, with the favorable allele for increased shelling percentage originating from teosinte. Specifically, the GZL2025_Rep2 and BLUP localization intervals were 142.91–162.46 Mb and 160.07–169.2 Mb, respectively. *qSP10* explained 2.96–6.45% of phenotypic variation across three replicates, with the favorable allele for increased shelling percentage originating from Zheng58. Specifically, the GZL2025_Rep1 localization interval was 121.27–125.79 Mb, GZL2025_Rep2 localization interval was 121.27–130.74 Mb, and BLUP localization interval showed a shift compared to the previous two replicates, ranging from 90.41 to 97.67 Mb. The detailed mapping results for qSP7 and qSP10 across the two field replicates and the BLUP analysis are summarized in Table 1.

### 3.4. Identification of Candidate Genes for qSP7 and qSP10

**Figure 5:**
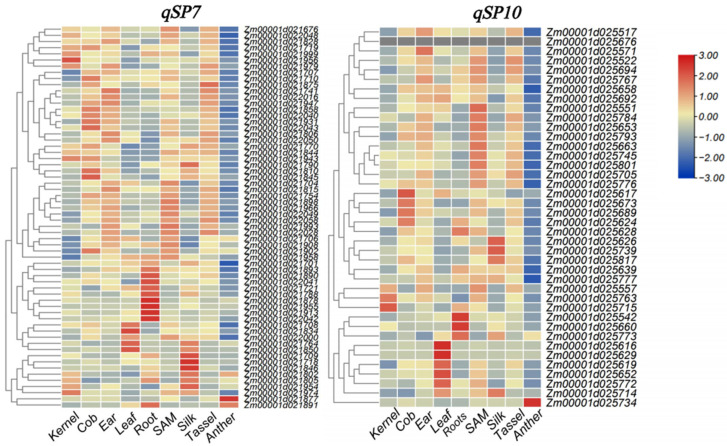
Relative expression in 9 tissues in *qSP7* and *qSP10*.

To identify candidate genes for *qSP7* and *qSP10*, all annotated genes within the corresponding QTL support intervals were first retrieved based on the B73 RefGen_v4 genome annotation. Their expression levels in V18 immature cob and whole seed at 22 days after pollination (22 DAP) were then examined using qTeller (MaizeGDB). Genes with expression values below 5 FPKM in both tissues were removed. The genes retained after this initial screening were further evaluated using publicly available transcriptome data [33], and their relative expression patterns across nine tissues (kernel, cob, ear, tassel, leaf, root, silk, anther, and SAM) are shown in Figure 5.

For *qSP7*, the QTL peak was consistently detected in the 160+ Mb region. Based on the support interval identified in GZL2025_Rep1 (160.07–169.20 Mb), 62 genes remained after the initial qTeller-based filtering. Their relative expression patterns across the nine tissues are shown in Figure 5. Among them, *Zm00001d021701*, encoding a basic helix–loop–helix (bHLH) transcription factor, was retained as a candidate gene. Members of the bHLH family have been implicated in maize kernel development, and the related gene *O11* has been reported to function in endosperm development [34]. Another retained candidate gene was *Zm00001d021708*, which encodes a pentatricopeptide repeat (PPR) superfamily protein. PPR proteins have also been reported to be associated with seed development and kernel-related traits [14,35,36,37,38,39].

For *qSP10*, the intervals detected in GZL2025_Rep1 and GZL2025_Rep2 overlapped substantially. Based on this shared region, the interval used for candidate gene screening was defined as 121.273–130.74 Mb. In contrast, the interval identified from the BLUP analysis was clearly shifted relative to the replicate-supported region and was therefore treated more cautiously in the subsequent interpretation. Within the replicate-supported interval, 40 genes remained after the same qTeller-based filtering procedure, and their relative expression patterns are also shown in Figure 5. Among these genes, *Zm00001d025739*, encoding a protein kinase superfamily protein, was retained as a candidate gene. This gene showed relatively high expression in kernel and cob, whereas its expression was much lower in silk and SAM, suggesting a possible relevance to shelling percentage.

Based on their positions within the QTL support intervals, expression patterns in relevant tissues, and annotated or reported functions, *Zm00001d021701*, *Zm00001d021708*, and *Zm00001d025739* were identified as candidate genes for qSP7 and *qSP10*, as shown in Table 2.

## 4. Discussion

Shelling percentage is a composite trait determined by the relative contribution of kernel biomass to total ear biomass, and is therefore influenced by multiple developmental processes, including kernel filling, cob growth, and ear structure [1,2]. Compared with kernel weight, kernel size, and related yield components, the genetic basis of shelling percentage has received relatively limited attention in maize [3,4]. In the present study, using a maize–teosinte BC_2_S_2_ population with strong phenotypic contrast, we identified two reproducible QTLs, *qSP7* and *qSP10*, associated with shelling percentage. Because teosinte and modern maize differ markedly in ear and kernel morphology, this population provided a useful genetic background for dissecting variation in this trait [26].

The phenotypic variation explained by *qSP7* and *qSP10* was relatively modest, ranging from approximately 3% to 6% across the two field replicates and the BLUP-based analysis. This is not unexpected for shelling percentage, which is a typical quantitative trait likely controlled by multiple loci of small effect and influenced by environmental conditions. For such traits, moderate- or small-effect QTLs can still be informative, especially when they are detected repeatedly across analyses. Therefore, the significance of *qSP7* and *qSP10* lies not only in their individual effect sizes, but also in their reproducibility and their potential to provide entry points for further dissection of the genetic basis of shelling percentage.

One interesting result of this study is that the favorable allele of *qSP7* was contributed by teosinte, whereas the favorable allele of *qSP10* was contributed by Zheng58. At first glance, the positive effect of a teosinte allele on shelling percentage may appear counterintuitive, given the low overall shelling percentage of teosinte [26]. However, such a pattern is not unusual for complex traits. The overall phenotype of a parent reflects the combined effects of many loci and does not exclude the presence of favorable alleles at individual loci. In highly divergent populations such as maize–teosinte crosses, alleles from the phenotypically inferior parent may still have positive effects in a maize genetic background. This result suggests that shelling percentage can be decomposed into locus-specific effects contributed by both parents and also highlights the value of teosinte germplasm as a source of useful allelic variation.

For *qSP10*, the support intervals identified in the two field replicates overlapped substantially, whereas the interval detected using BLUP values was shifted relative to the replicate-supported region. Because BLUP integrates information across replicates, such displacement may reflect the combined effects of modest QTL effect size, environmental variation, and the limited resolution of the linkage map. In particular, the presence of relatively large marker gaps in some genomic regions may have reduced interval precision and contributed to instability in peak location. For this reason, in the current study we placed greater emphasis on the region consistently supported by the two field replicates, while treating the BLUP-derived interval more cautiously in the subsequent interpretation. This is a more conservative interpretation of the *qSP10* signal and better reflects the current mapping resolution.

The linkage map generated in this study provided genome-wide coverage for QTL detection, but its resolution remains limited for fine-scale inference. As shown by the distribution of markers across chromosomes, marker density was not uniform throughout the genome, and some intervals were more sparsely covered than others. This limitation should be taken into account when interpreting both QTL boundaries and candidate genes. Accordingly, the genes highlighted here should be regarded as interval-based candidates rather than genes directly supported by high-resolution SNP-level evidence.

Within the support intervals of *qSP7* and *qSP10*, three genes were highlighted as plausible candidates. For *qSP7*, *Zm00001d021701*, encoding a bHLH transcription factor, is of particular interest because transcription factors in this family have been implicated in maize kernel development [20], and the related gene *O11* has been reported to function in endosperm development and nutrient metabolism [34]. *Zm00001d021708*, encoding a PPR superfamily protein, was also retained as a candidate because PPR proteins are frequently associated with seed development and kernel-related traits [14,35,36,37,38,39]. For *qSP10*, *Zm00001d025739*, annotated as a protein kinase superfamily protein, was prioritized because kinase-mediated pathways are widely involved in developmental regulation, and signaling components associated with kernel filling and seed development have been reported in maize [34,40,41]. Nevertheless, these genes should still be regarded as candidate genes rather than validated causal genes. Their possible roles in shelling percentage remain tentative and will require further confirmation through finer mapping and functional validation.

From a breeding perspective, the QTLs identified here are more appropriately viewed as preliminary genetic resources than as immediately deployable markers. Their modest effects suggest that direct application in marker-assisted selection should be approached cautiously, particularly before validation in additional genetic backgrounds. Even so, these loci provide useful starting points for future work on shelling percentage and may contribute to marker development after further verification. More broadly, this study expands current knowledge of the genetic architecture of shelling percentage in maize and provides a basis for future efforts to connect ear structure, kernel development, and harvest-related traits at the genetic level.

## 5. Conclusions

Using a maize–teosinte BC_2_S_2_ population, this study identified two reproducible QTLs, *qSP7* and *qSP10*, associated with shelling percentage in maize. These results indicate that shelling percentage is a complex quantitative trait controlled by multiple loci with modest effects. Several candidate genes were identified within the *qSP7* and *qSP10* intervals based on genomic position, expression pattern, and functional annotation, but they remain putative candidates requiring further validation. Overall, this study provides a basis for future genetic analysis and marker development for shelling percentage in maize.

## Figures and Tables

**Table 1:** QTL mapping results of shelling percentage in three replicates.

QTL	Environment	Chr	LOD ^a^	Var (%) ^b^	Add ^c^	Peak (Mb) ^d^	Support Interval (Mb) ^d^
*qSP7*	GZL2025_Rep1	7	4.1	3.41	0.03	166.93	160.07–169.2
*qSP7*	GZL2025_Rep2	7	4.99	3.35	0.04	162.17	142.91–162.46
*qSP7*	BLUP	7	4.91	3.39	0.03	161.62	142.91–162.46
*qSP10*	GZL2025_Rep1	10	5.9	4.56	−0.07	122.71	121.27–125.79
*qSP10*	GZL2025_Rep2	10	6.84	2.96	−0.06	129.06	121.27–130.74
*qSP10*	BLUP	10	6.83	6.45	−0.06	93.91	90.41–97.67

^a^ LOD means logarithm of odds. ^b^ Var (%) indicates the percentage of phenotypic variation explained by QTLs. ^c^ Positive or negative addictive effect values indicates that the alleles from teosinte or Zheng58 increased the phenotypic value, respectively. ^d^ (Mb) is based on the B73 v4 reference genome.

**Table 2:** Functional annotation of potential candidate genes.

QTL	Chr	Candidate Gene ID	Function Annotation
*qSP7*	7	*Zm00001d021701*	Basic helix–loop–helix (bHLH) DNA-binding superfamily protein
*qSP7*	7	*Zm00001d021708*	Pentatricopeptide repeat (PPR) superfamily protein
*qSP10*	10	*Zm00001d025739*	Protein kinase superfamily protein

## Data Availability

The main datasets supporting the conclusions of this article are included within the article and its additional file. The RNA-seq datasets used in this study are available in this reference: Yi, F.; Gu, W.; Chen, J.; Song, N.; Gao, X.; Zhang, X.; Zhou, Y.; Ma, X.; Song, W.; Zhao, H.; et al. High Temporal-Resolution Transcriptome Landscape of Early Maize Seed Development. *Plant Cell* **2019**, *31*, 974–992, https://doi.org/10.1105/tpc.18.00961 [33].

## Supplementary Materials

The following supporting information can be downloaded at https://www.mdpi.com/article/10.3390/genes17040384/s1. Table S1: Broad-sense heritability and descriptive statistics for shelling percentage in the TM population across two replicates; Table S2: Summary statistics of the genetic linkage map constructed for the TM population, including marker number and map length for each chromosome.
